# Code Blue Emergencies: A Team Task Analysis and Educational Initiative

**Published:** 2012-03-31

**Authors:** James W. Price, Oliver Applegarth, Mark Vu, John R. Price

**Affiliations:** 1 Department of Anesthesiology, Pharmacology & Therapeutics, Vancouver General Hospital; 2 University of British Columbia, Vancouver, British Columbia, Canada

## Abstract

**Introduction:**

The objective of this study was to identify factors that have a positive or negative influence on resuscitation team performance during emergencies in the operating room (OR) and post-operative recovery unit (PAR) at a major Canadian teaching hospital. This information was then used to implement a team training program for code blue emergencies.

**Methods:**

In 2009/10, all OR and PAR nurses and 19 anesthesiologists at Vancouver General Hospital (VGH) were invited to complete an anonymous, 10 minute written questionnaire regarding their code blue experience. Survey questions were devised by 10 recovery room and operation room nurses as well as 5 anesthesiologists representing 4 different hospitals in British Columbia. Three iterations of the survey were reviewed by a pilot group of nurses and anesthesiologists and their feedback was integrated into the final version of the survey.

**Results:**

Both nursing staff (n = 49) and anesthesiologists (n = 19) supported code blue training and believed that team training would improve patient outcome. Nurses noted that it was often difficult to identify the leader of the resuscitation team. Both nursing staff and anesthesiologists strongly agreed that too many people attending the code blue with no assigned role hindered team performance.

**Conclusion:**

Identifiable leadership and clear communication of roles were identified as keys to resuscitation team functioning. Decreasing the number of people attending code blue emergencies with no specific role, increased access to mock code blue training, and debriefing after crises were all identified as areas requiring improvement. Initial team training exercises have been well received by staff.

## Introduction

The operating room nurses at Vancouver General Hospital have recently implemented a program of nursing education days. The purpose of this initiative was for nurses to identify areas of their practice which require further training. Prior to this, an informal survey of operating room (OR) and post-anesthesia recovery room (PAR) nursing staff at Vancouver General Hospital identified code blue situations as the most stressful situations that nurses face in their daily practice. Therefore, this survey also identified an area of our anesthesiology practice that required improvement. In discussions with the department of nursing and anesthesiology, we decided to create a questionnaire to gather information on the current state of code blue management in the OR and PAR from the perspective of both groups of health professionals.

Our questionnaire was developed after a thorough literature search revealed little background information with respect to interdisciplinary code blue management in the OR and PAR. Although there is substantive evidence suggesting better performance by medical trainees (medical students and residents) during simulated code blue scenarios and other complex procedural tasks after high-fidelity simulation training sessions, no previous studies have asked resuscitation team members in the operating room and post-anesthesia recovery rooms (nursing staff and anesthesiologists) what they believe are the essential components of effective team performance during a code blue resuscitation.^1^,^2^

The purpose of this study was to identify both positive and negative factors affecting code blue management in the OR and PAR at a major Canadian teaching hospital and then to address these deficits with an ongoing educational initiative aimed at improving code blue team performance in our OR and PAR units.

## Methods

### Survey Development

The literature search was limited to human and English-language articles. MEDLINE and CINAHL (1966 to September 2010) were searched with the terms ‘nursing’, ‘code blue’, ‘crisis resource management’, ‘operating room’, ‘anesthesiologists’ and permutations thereof. Search terms were selected through discussion with nurses, anesthesiologists, and librarians with considerable medical education and simulation teaching experience, therefore suggesting that these terms would capture inclusive results. Hand searching references from papers collected and internet searching were also employed. To our surprise, no studies were identified which addressed code blue training or management in the operation room and post-anesthesia recovery room from nurses’ or anesthesiologists’ perspectives.

Two separate surveys were developed for nursing staff and anesthesiologists regarding their experience with code blue emergencies. Both questionnaires were rigorously designed and evaluated prior to administration. Ten recovery room and operating room nurses as well as five anesthesiologists who had an interest in medical education were contacted by the authors prior to survey creation. These professionals represented four different hospitals in British Columbia (years of experience ranging from 5–30 years), and each contributed questions for the survey. Questions were compiled and three iterations of the survey were reviewed by this pilot group of nurses and anesthesiologists and their feedback was integrated into the final version of the survey. To compensate survey participants for their time and to increase survey response rate, two gift certificate prizes were available for those who completed the survey (total value $200).

The survey used likert-scale multiple-choice responses combined with open-ended questions. A scaled rating methodology was selected, as this was similar to previous studies that assessed medical education environments.^3^,^4^ Respondents were asked to indicate their agreement with each statement ranging from strongly disagree to strongly agree. Responses included: strongly disagree (1), disagree (2), neutral (3), agree (4), and strongly agree (5). The questionnaires can be found in Appendix 1 and 2.

Participants were then invited to complete the 10-minute, anonymous survey (41 questions for nursing staff, 35 questions for anesthesiologists, which were made available for 8 weeks. Two sets of reminder e-mails were sent to both nurses and anesthesiologists at 3-week intervals. All data were stored within a locked cabinet on-site at Vancouver General Hospital and were subsequently transcribed onto a password-protected departmental computer for analysis.

### Data Analysis

SPSS 8 (SPSS Inc., Chicago, IL, USA) was used for statistical analysis. Multiple choice questions are presented as median (interquartile range). For open-ended questions, themes were discovered through data analysis and inter-rater reliability testing was performed to ensure at least 80% agreement between the three separate individuals, who categorized the data and identified themes. The ‘Top 3’ responses and the number of times they appeared in the data were reported where applicable. Numerical data, such as the participants’ age, number of years worked and the number of code blues they participated in are presented as means.

## Results

### Demographics

A total of 49 nurses (OR and PAR combined) and 19 anesthesiologists responded to our survey which represents a response rate of 37.7% and 37.2% respectively (of a total of 70 full time OR nurses, 60 full time PAR nurses, and 51 anesthesiologists). This was in keeping with the response rate of previous surveys of resident doctors in Canada at 27.4% and a review of 199 online surveys from a variety of disciplines indicating a comparable average response rate of 32.5%.^3^,^4^ The demographic data are shown in Table 1.

All nursing staff strongly supported mandatory code blue training and believed that it would improve both patient outcome and their own comfort in managing code blue situations (Table 2). Very few nurses had previously participated in code blue training, had completed advanced cardiac life support (ACLS) training, or had used a patient simulator for learning (Table 3). Nursing staff felt anxious about participating in code blue training with their colleagues watching, but more comfortable if their colleagues would participate alongside them in code blue training exercises. Nursing staff strongly supported using a debriefing process following code blue emergencies, but noted that this rarely happens in their daily practice. With respect to roles during code blue emergencies, nursing staff were most comfortable acting as the events recorder, followed by acting as the drug administration nurse. Nursing staff were least comfortable controlling the defibrillator.

Nurses strongly agreed that the the anesthesiologist should be the leader of the code blue. However, nurses in both the operating room and recovery room noted that in many of their experiences it was often difficult to identify the leader of the code. Nursing staff noted that anesthesiologists needed to improve on announcing the drugs being administered during crisis situations so that the recording nurse could document them accurately. Interestingly, most anesthesiologists felt that they clearly announced the drugs given during the code blue to the recording nurse.

### Anesthesiologists

In contrast to what was noted by nurses, anethesiologists said that they clearly announced their role and were easy to identify as the leader of code blue situations in the OR and PAR. Anesthesiologists believed that debriefing following a code blue took place more often than nursing staff reported and fully supported the use of debriefing after a crisis situation. Few anesthesiologists had attended code blue training sessions or had used patient simulators for learning. Anesthesiologists also suggested that nurses needed to improve the communication of their role to the team leader during a crisis.

Both nursing staff and anesthesiologists strongly agreed that too many people attending the code blue with no assigned role was a real issue that needed to be addressed in both the operating room and post-operative recovery unit. Both groups agreed that “code blues are effectively run at our hospital”.

## Discussion

The results of this questionnaire provide insight into factors influencing the most critical, life-threatening situations in the OR and PAR: code blue resuscitations. Areas that were singled out as critical for optimal performance in a code blue scenario were: effective leadership with clear communication between team members, coordinated team functioning, and crowd control. Post-resuscitation debriefing was also identified as an area requiring improvement.

### Leadership and Communication

An effective leader is of paramount importance in the functioning of the code blue team.

A recent study noted that a 10-minute period of instruction in leadership skills improved resuscitation skills of medical students in a simulated code blue environment.^2^ These instructions not only improved resuscitation skills but also led to more rapid and sustained CPR performance – a factor which has been stressed as one of the most important changes in the revised ACLS guidelines. Leadership instruction included: deciding what to do, telling your colleagues what they should do, making short, clear statements, and ensuring adherence to the ACLS algorithm.

One suggestion to help improve leadership and communication from the anesthesiologist during crises may come from the ‘SBAR’ pneumonic, which is used on hospital wards for patient handover and communicating critical patient information between healthcare professionals.^7^,^8^ The letters ‘SBAR’ stand for: Situation, Background, Assessment, and Recommendations, and is well known in the medical education literature. In contrast to previous models using ‘SBAR’ for handing over care for patients, we would suggest that anesthesiologists could use this technique in an emergency situation as follows:

After inducing a patient in the operating room and noting profound hypotension and lack of a pulse the anesthesiologist announces to the operating room:

Situation: *“I need everyone’s attention, we have a life threatening emergency here, Patrick (nurse) please call a code blue immediately.”*

Background: *“We have a 53 year old man who presented with a perforated viscous in the emergency department who is now in a pulses electrical activity arrest post-induction.”*

Assessment: *“I think this is likely due to the patient’s overwhelming sepsis and a result of a relative anesthetic overdose with induction.”*

Recommendation: “*Dr. Keegan (staff surgeon) and Dr. Coppin (resident surgeon) please start CPR immediately while I administer a fluid bolus and draw up epinephrine to support his blood pressure….”*

This approach has the three-fold effect of increasing situational awareness among team members: establishing a chain of command, clearly identifying the anesthesiologist as the resuscitation leader, and outlining a plan with identified team members performing specifically delegated tasks. As noted in our survey, operating room nurses believed that poor communication resulted from anesthesiologists assuming that nurses know what was the cause and treatment of the arrest, where this was often not the case. The regular use of ‘SBAR’ throughout the resuscitation would ensure that nursing staff not only understand their role in resuscitation but also the code leader’s assessment and intended plan of action – all identified in our survey as being keys to successful team performance during resuscitation.

With respect to educational modality, nurses strongly supported simulation as the preferred modality to practice code blue training, whereas anesthesiologists noted both case-based learning and simulation equally as the preferred learning tool. Previous studies have suggested that “anxiety about performing in front of peers is the largest hurdle for anesthesiologists participating in simulation-based training exercises”.^9^

### Coordinated Team Function and Team Training

Training is defined as the acquisition of knowledge, skills and behaviours that lead to an improvement in performance in a particular domain. Salas et al. completed an extensive, cross-disciplinary meta-analysis examining whether team training translates into improved team performance. Using rigorous inclusion criteria, 45 primary studies were included in the analysis, which included a total of 2650 teams from such diverse backgrounds as the military, aviation, and the business sector.^10^ Eighty of the teams included in the review were from the field of medicine. The findings of the analysis suggest that team training accounted for approximately 12% to 19% of the variance in examined outcomes. Salas et al. suggest that this was likely an underestimate of the benefit of team training on subsequent performance. Salas et al. go on to note that: “given the heightened interest in team training in health care, change agents in health care institutions should utilize this information to bolster their argument for implementing such training.”

### Nurse Training

A comprehensive review of the literature was performed to assess nurses’ knowledge and skill retention following cardiopulmonary resuscitation training.^11^ Twenty-four studies met inclusion criteria and were included in the final results. The results indicate that nurses benefited from practicing commonly seen arrest scenarios using simulation.

Current evidence supports the need for ACLS training for all critical care nurses. Previous studies also have demonstrated that skill and knowledge degradation is common and to keep skills effective and patients safe, an ongoing training program for resuscitation teams is essential among nurses.^12^ It has been suggested that the use of ‘surprise’ mock codes are key to improving team performance during actual emergency situations both at a departmental and hospital level.^14^

### How to Get Started with Team Training: Team Task Analysis

**Figure 1:**
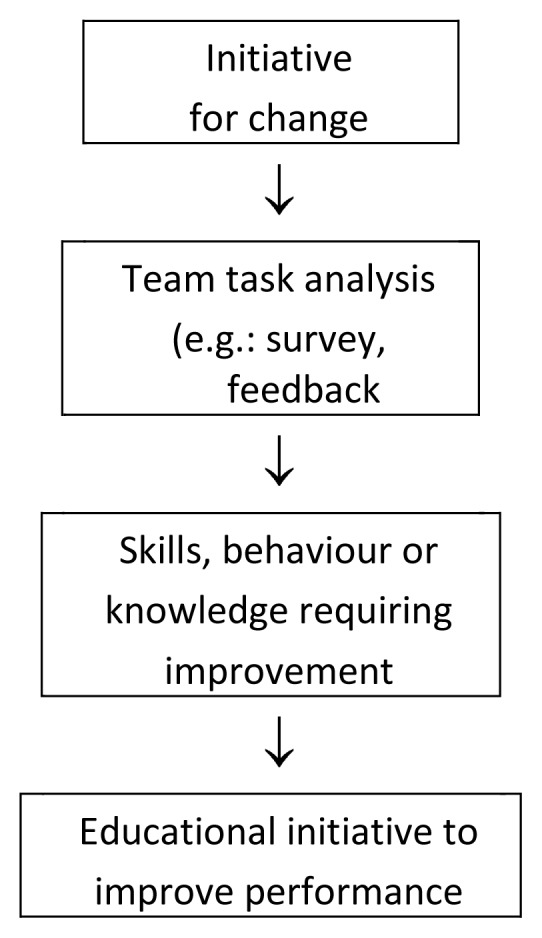
Educational Initiative Flow Chart.

Team task analysis is a procedure for determining the operational skills needed for the smooth coordination of a team.^14^ After identification of these components, the team can then practice and learn the requisite knowledge, skills and behaviours necessary to improve performance (Figure 1).

Our questionnaire can be thought of as a team task analysis for OR and PAR nurses and anesthesiologists with respect to team training and code blue management at our hospital. The training that follows this initial fact-finding survey should incorporate guidelines for effective team training which include: pre-practice tools, emphasis on teamwork components identified in the team task analysis, ensure that training facilitates adaptive behaviours, promotes a safe learning climate where team members can voice their opinion freely, ensures team members apply closed loops of communication. and is followed by a post-training evaluation of the training intervention.^14^

As noted in our survey findings, most nurses and doctors cite ‘time pressure’ as the biggest hurdle to their participation in code blue training sessions. Therefore, to improve delivery of team training to nurses and anesthesiologists, sessions should be built into nurses’ and doctors’ continuing education and mandatory rounds time. This has been accomplished at the authors’ institution by scheduling mock codes in the OR and PAR during weekly anesthesiology and nursing rounds. Anesthesiologists volunteer to lead mock codes in the company of an anesthesiologist moderator and an experienced OR or PAR nurse. Sessions are built upon a safe, no-fault learning environment and the use of repetitive practice of commonly encountered emergencies and procedures in each unit. Each session is learner-centred with sufficient time to complete a debriefing process to help plan future mock code blue training sessions.

### Crowd Control

Having too many responders in the OR and PAR during code blue resuscitation was identified by both nurses and anesthesiologists as hindering team performance. In a previous study of 30 hospital ward nurses, too many individuals present at resuscitations was identified as a significant barrier to effective team functioning.^15^ There is currently little data in the literature on how large crowds of onlookers with no assigned role at code blue resuscitations may affect team performance and ultimately patient outcome.

Suggestions to help with crowd control in our questionnaires included restricting the number of people allowed to enter the operating room, creating a daily code blue team, and self-awareness education whereby people attending code blues who are not directly involved in the resuscitation effort could help to reduce the number of people who are acting as a distraction to the resuscitation team by removing themselves and others from the area.

### Debriefing

The use of debriefing as a learning tool was seen to show dramatic improvements in team performance of an operating room team learning a new method of minimally invasive cardiac surgery.^16^ Given all the educational, emotional, and team building benefits of effective debriefing, it was surprising to find that both nursing staff and anesthesiologists rarely participated in debriefing sessions after code blue emergencies (Table 2). Both groups would like to see debriefing sessions occur more regularly. Collecting all team members together and organizing a team debriefing session should be the responsibility of the team leader and this is something that we have started to employ at our institution. As with all feedback, feedback during debriefing sessions should be courteous, relevant for the learner, given in manageable amounts, and solicited from all team members.

### Future Directions

At our institution, we have used the results of this survey to plan monthly, multidisciplinary mock code blue training sessions for our anesthesiologists, OR and PAR nurses that are relevant to our daily practice and educational needs. We invite respiratory therapists, surgeons and residents to participate when they are available. Using the feedback we have received in our survey, our scenarios and mock code blue sessions aim to improve the anesthesiologist’s announcement of taking the leadership role during the resuscitation, the support of this leadership role by other anesthesiologists attending the resuscitation, the use of the ‘SBAR’ technique at the beginning, middle, and end of the emergency, improving communication between code blue team members, and having strategies to ensure the ideal number of people respond to each resuscitation. We are hoping to collect data on this educational initiative as it progresses, so that staff can be instrumental in the future direction of our mock code blue program.

### Limitations of the Survey

Completion of the questionnaire was on a voluntary basis and, therefore, may have biased the results toward the feedback of more motivated participants.

It is also important to note that this survey only sought feedback from nursing staff and anesthesiologists. In an emergency situation many healthcare professionals come together to form a multi-sisciplinary team. Expanding this survey to include surgical staff, respiratory and anesthesiology technical staff and OR/PAR attendant staff could be looked at in a future study. As institutions develop their own mock code blue training programs it would be important to include these groups in the training sessions as doing so would simulate the entire code blue team and help create a training program that addresses all code blue team members’ educational requirements.

### Conclusion

A 2004 joint commission report in the United States for hospital accreditation noted ‘communication failure’ as the primary cause for inadvertent patient harm in the hospital.^17^ Moreover, communication errors played a role in 75% of all fatal events in hospital. Our study demonstrates that nurses and anesthesiologists believe that instituting ongoing, multi-disciplary team training sessions for those professionals involved in code blue resuscitations leads to improved team performance. Improving resuscitation team performance will ultimately help the sickest patients in the hospital when they need it most.

## Figures and Tables

**Table 1:** Demographic data, code blue experience and ideal number of practice sessions per year of OR/PAR nurses and anesthesiologists.

Question	OR Nurses (n = 22)	PAR Nurses (n = 25)	Anesthesiologists (n = 19)
Gender	20 female, 2 male	23 female, 2 male	2 female, 17 male
Age	44.7 (14.0)	41.7 (11.1)	49.1 (10.4)
Years experience	11.6 (8.77)	11.1 (10.2)	16.4 (10.1)
No. of codes involved with	20.7 (25.4)	10.8 (19.9)	34.5 (38.0)
Ideal number of code blue practice sessions/yr	1.64 (1.10)	1.75 (0.91)	1.10 (0.83)

**Table 2:** Survey Responses.*

Question	OR Nurses (n = 22)	PAR Nurses (n = 25)	Anesthesiologists (n = 19)
1. I have a clear understanding of my role during a code blue	4 (1)	4 (1)	4 (2)
2. I feel comfortable announcing my role and communicating with the resuscitation team during a crisis	4 (1)	4 (1)	4 (1)
3. The team can easily identify that the anesthesiologist is in charge during code blues	3 (2)	3 (2)	4 (1)
4. The anesthesiologist should be in charge of running the code blue	5 (1)	5 (1)	N/A
5. The effectiveness of chest compressions is clearly being assessed in most cardiac arrest situations	3 (1)	4 (1)	4 (1)
6. I believe one nursing role during code blues should be crowd control	3 (2)	4 (1)	4 (1)
7. I believe one nursing role during code blues should be assessing the effectiveness of chest compressions during cardiac arrest	3 (1)	3 (1)	2 (1)
8. I feel comfortable drawing up resuscitation drugs during a code blue	4 (1)	3 (2)	N/A
9. I feel comfortable acting as the events recorder during a code blue	4 (0)	3 (1)	N/A
10. I feel comfortable operating the defibrillator during a code blue	3 (2)	4 (2)	4 (1)
11. While taking part in a code blue, I feel comfortable asking for help	4 (1)	4 (0)	5 (1)
12. During a code blue I am most concerned about making a mistake	3 (2)	3 (2)	2 (1)
13. The code leader / I clearly announce the drugs I administer during a code blue	2 (2)	2 (1)	4 (1)
14. Crowd control is an issue at code blues in the OR/PAR	4 (1)	4 (1)	4 (1)
15. The use of patient simulators could play an important role in my critical incident training	4 (1)	5 (1)	4 (0)
16. Code blue training should be multi-disciplinary, including nursing, anesthesiology, and surgery	4 (1)	5 (1)	4 (1)
17. Practicing multi-disciplinary, team-based code blue scenarios at my institution would make me more comfortable in code blue situations	4 (1)	5 (1)	4 (0)
18. Practicing multi-disciplinary, team-based code blue scenarios at my institution would improve patient outcomes	4 (1)	4 (1)	3 (1)
19. Code blue and crisis management training should be a mandatory part of my continuing education	5 (1)	5 (0)	4 (0)
20. I feel anxious about participation in mock code blue scenarios	3 (2)	3 (1)	2 (1)
21. I would feel comfortable participating in a code blue scenario with my colleagues observing me	4 (1)	4 (2)	3 (1)
22. I would feel comfortable participating in a code blue scenario with my colleagues also participating alongside me	4 (0)	4 (1)	4 (1)
23. After a code blue, the team involved undergoes a debriefing process recapping the events and allowing all team members to voice concerns	1 (1)	2 (2)	2 (1)
24. I believe that team debriefing after code blues is important	4 (1)	5 (1)	4 (0)
25. I believe that code blues in the OR/PAR at VGH are effectively run	4 (1)	3 (1)	4 (1)

* Reported as median (interquartile range) according to 5 point Likert scale (1 = strongly disagree, 2 = disagree, 3 = neutral, 4 = agree, 5 = strongly agree). N/A = question not asked.

**Table 3:** Crowd control, previous code blue training, and the use of simulation for education.

Statement	OR Nurses n = 22	PAR Nurses n = 25	Anesthesiologists (n = 19
In my experience there are too many people in the room during code blues	95.5%	80%	84.2%
I have completed previous code blue training	45.5%	92%	36.8%
I have previously used a patient simulator for code blue training	13.6%	72%	42.1%

* Responses dichotomized and reported as percentage of responses indicating “agree” and “strongly agree” with the respective statements.

**Table 4:** Open-ended Responses. (Top 3 responses reported with number of responses in brackets)

Question	OR Nurses	PAR Nurses	Anesthesiologists
**The reason for poorly run codes at my hospital are:**	1. Poor communication (“MD’s assumed we knew what was going on”) (10)	1. No leader identified (17)	1. Too many people in the room/Patient factors /Unclear leader (12)
	2. Poorly defined roles for team members (5)	2. Too many people in the room (15)	2. Poor communication (4)
	3. Too many people in the room (5)	3. No role designation (2)	3. Fixation errors/poor role delegation (3)
**My anxiety during a code blue comes from:**	1. Lack of training, not knowing what to do (10)	1. Lack of training, not knowing what to do (11)	1. Patient’s outcome (7)
	2. Not knowing who to listen to (7)	2. Performance anxiety (6)	2. Concern over reason for code being a personal error (3)
	3. Performance Anxiety/too	3. Too many people in room/angry doctor shouting orders (5)	
**How to improve code blue team performance at my hospital:**	1. More training sessions (22)	1. More training sessions/mock codes (21)	1. More training sessions/mock codes (7)
	2. Improving communication skills among team members/better leadership from anesthesiologist (7)	2. Improve leadership from anesthesiologist (8)	2. Decreased number of people in the room (6)
	3. Crowd control/more debriefing (6)	3. Crowd control/more debriefing (6)	3. Better leadership/communication/role identification (3)
**The best modality of the code blue team to practice and learn is:**	1. Simulation (20)	1. Simulation (20)	1. Simulation (8)
	2. Case-based learning (8)	2. Case-based learning (10)	2. Case-based learning (8)
**Hurdles to my participation in code blue training sessions are:**	1. Time constraints (19)	1. Time constraints (15)	1. Time constraints (9)
	2. Training is not available (4)	2. Performance anxiety/Monetary compensation (5)	2. Performance anxiety (2)
	3. Monetary compensation (4)		
**Nurses could help team performance during code blues by:**	N/A	N/A	1. Improved communication of their roles to team leader (12)
			2. Improved documentation (5)
			3. More training mock codes (2)

## APPENDIX 1 VGH OR/PAR Nursing Code Blue Questionnaire

Please help us assess the experience of VGH OR & PAR nurses with respect to code blues

All responses will be kept confidential and you may withdraw your participation at anytime

## APPENDIX 2 VGH Anesthesiologist Code Blue Questionnaire

Please help us assess the experience of VGH Anesthesiologists with respect to code blues

All responses will be kept confidential and you may withdraw your participation at anytime
